# Short-term thinning alters labile soil phosphorus fractions and is associated with microbial alpha diversity and phosphatase activity in subtropical conifer plantations

**DOI:** 10.3389/fpls.2026.1926759

**Published:** 2026-08-18

**Authors:** Bingxu Yang, Long Li, Kaiyang Luo, Shijie Li, Yubing Liu, Kang Lin, Laicong Luo, Xintong Xu, Lingjian Tao, Yuanqiu Liu

**Affiliations:** 1 College of Forestry, Jiangxi Agricultural University/Jiangxi Provincial Key Laboratory of Subtropical Forest Resources Cultivation, Nanchang, China; 2 Lushan Forest Ecosystem Positioning Observation and Research Station, Jiujiang, China

**Keywords:** Chamaecyparis obtusa, Cryptomeria japonica, Hedley phosphorus fractions, microbial alpha diversity, phosphatase, soil phosphorus availability, subtropical conifer plantations, thinning

## Abstract

**Introduction:**

Plantations play important roles in ecosystem functions but long-term high-density management can limit nutrient cycling. Phosphorus is a key limiting nutrient in subtropical forest soils due to strong fixation by Fe/Al oxides. The Hedley fractionation method provides detailed insight into soil P pools, especially labile and moderately labile fractions. Soil microorganisms and phosphatase enzymes are important regulators of P transformation. However, subtropical introduced conifers + paired first-year responses + Hedley fractions + microbial/enzymatic associations. This study investigates thinning effects on P fractions, microbial diversity, and phosphatase activity in two conifer plantations.

**Methods:**

Study was conducted in Lushan National Nature Reserve, Jiangxi Province, China. Japanese cedar and Hinoki cypress plantations were used. Treatments included control, light, moderate, and heavy thinning. Soil samples were collected from 0–10 cm and 10–20 cm layers before and one year after thinning. Soil P fractions were measured using Hedley sequential extraction. Microbial diversity was analyzed using 16S rDNA and ITS sequencing. Phosphatase activity was measured using p-nitrophenyl phosphate method. Statistical analyses included ANOVA and redundancy analysis (RDA).

**Results:**

Thinning significantly increased labile P and moderately labile P fractions, especially in surface soils. Thinning-related changes were observed in both Japanese cedar and Hinoki cypress plantations, although their magnitudes varied among thinning treatments. Microbial alpha diversity and acid phosphatase activity generally increased under thinning, although responses varied among treatments. RDA showed strong coupling between labile P fractions, microbial diversity, and phosphatase activity, while stable P fractions remained largely unchanged.

**Discussion:**

Thinning-related increases in labile P fractions were associated with changes in microbial alpha diversity and phosphatase activity, indicating that microbial and enzymatic indicators were associated with thinning-related changes in soil P fractions. Increases in labile and moderately labile P fractions reflect changes in operationally defined active P pools rather than detectable changes in total P. The observed associations are consistent with the known roles of microbial communities in decomposition and of acid phosphatase in potential organic P mineralization. Species-specific differences indicate that stand structure and litter quality regulate the magnitude of thinning effects. Overall, the coordinated responses of microbial diversity, phosphatase activity, and active P fractions are consistent with a potential microbial–enzyme–P coupling mechanism.

## 1 Introduction

Plantations play key roles in timber production, soil and water conservation, ecological restoration, and carbon sequestration and are therefore important components of forest management and ecosystem functioning (Liang et al., 2016). However, long-term high-density monoculture can simplify stand structure, restrict understory vegetation development, weaken soil nutrient cycling, and reduce biodiversity, thereby compromising plantation ecosystem stability (Wang et al., 2021). Mount Lu is among the earliest regions in mainland China where Japanese cedar (*Cryptomeria japonica*) and Hinoki cypress (*Chamaecyparis obtusa*) were introduced. These plantations contributed substantially to early barren-hill afforestation, water conservation, and ecological restoration. With increasing stand age, however, some stands have developed excessive density, poor natural regeneration, soil acidification, and declining site fertility, which constrain further improvements in ecosystem services (Liu et al., 2023). Appropriate stand-structure regulation is therefore an important management priority for maintaining soil nutrient cycling and promoting the sustainable management of introduced conifer plantations on Mount Lu (Ali et al., 2019).

Thinning is widely used to regulate stand structure in plantation management. By reducing stand density and canopy closure, thinning modifies understory light, soil temperature and moisture, inter-tree competition, understory vegetation, litter decomposition, fine-root turnover, and rhizosphere processes. These changes may alter litter- and root-derived P inputs, plant P uptake, microbial activity, extracellular enzyme activity, and the transformation of P among soil fractions (Li et al., 2020; Zhang et al., 2023; Zhou et al., 2020). Previous studies in larch and pine plantations have shown that thinning can alter soil available P and operationally defined P fractions, including labile, moderately labile, and relatively stable P pools (Tian et al., 2019; Liu et al., 2018; Jian et al., 2025; Ye et al., 2018). However, the direction and magnitude of these responses have varied among thinning intensities, soil depths, plantation types, and recovery periods. These variable responses indicate that thinning does not uniformly increase soil P availability but may instead alter the balance among litter- and root-derived P inputs, plant P uptake, microbial immobilization and mineralization, and the redistribution of P among soil P pools. In addition, previous studies have often focused on a single plantation type, relied primarily on post-treatment comparisons, or examined soil P fractions, microbial communities, and extracellular enzyme activities separately (Xu et al., 2024). Consequently, paired pre- and post-thinning evidence that simultaneously evaluates active and relatively stable P pools and their associations with microbial and enzymatic indicators across plantation types and soil depths remains limited. Phosphorus is a key nutrient limiting forest productivity and ecosystem functioning. In subtropical acidic forest soils, P is strongly sorbed by Fe and Al oxides or incorporated into relatively stable mineral-associated pools, thereby reducing its potential availability to plants and microorganisms. Clarifying how different soil P pools respond to thinning, and whether these responses are associated with microbial diversity and phosphatase activity, is therefore important for understanding the biological regulation of soil P cycling under plantation management.

Compared with total P or available P alone, soil P fractions provide a more detailed indication of P-pool lability, transformation pathways, and potential supply capacity. The Hedley sequential extraction procedure separates soil P into operationally defined fractions that differ in extractability and potential bioavailability. H_2_O- or resin-extractable P and NaHCO_3_-extractable P are generally classified as labile pools, whereas NaOH-extractable P represents a moderately labile pool. In contrast, HCl-extractable and residual P are commonly considered relatively stable or less available pools (Rodionov et al., 2020; Chi et al., 2022). These operationally defined fractions can therefore be used to evaluate whether thinning is associated primarily with changes in active P pools or with broader changes in relatively stable P pools. Changes in NaOH-extractable P may reflect the accumulation or redistribution of P within an operationally defined, moderately labile pool that can include Fe/Al-associated P. However, changes in this fraction alone cannot demonstrate the activation or release of Fe/Al-bound P, because the mineral forms of Fe/Al oxides and the associated P sorption-desorption processes were not directly measured in the present study (Gypser et al., 2018).

Soil microorganisms are key biological drivers linking changes in stand structure to P transformation. By decomposing litter, mineralizing organic P, secreting phosphatases, and releasing organic acids, microorganisms can promote the release, transformation, and redistribution of soil P (Lull et al., 2024; Tian et al., 2019; Liu et al., 2021). Phosphatase activity reflects the potential for organic P mineralization and is an important indicator of soil P transformation. Although microbial communities and phosphatases are potentially relevant to soil P transformation, few thinning studies have evaluated short-term changes in bacterial and fungal alpha-diversity indices, potential acid and alkaline phosphatase activities, and Hedley P fractions within the same paired experimental design. Consequently, it remains unclear whether first-year changes in labile and moderately labile P are statistically associated with these biological indicators in subtropical introduced conifer plantations. Because alpha diversity and potential phosphatase activity do not directly quantify microbial P-cycling functions or *in situ* P mineralization, these measurements can identify associations but cannot establish causal biological pathways.

A further unresolved question is whether co-occurring plantation types respond differently to the same thinning-intensity gradient under comparable site conditions. Although Japanese cedar and Hinoki cypress are both typical introduced conifer plantation species on Mount Lu, they may differ in canopy structure, litter quality, root activity, and understory vegetation development. These differences may lead to contrasting responses of soil P fractions and microbial processes after thinning. Comparing the two plantation types within the same region and under the same thinning-intensity gradient can therefore help determine whether plantation type modifies thinning-related changes in soil P fractions and biological indicators and can provide a basis for differentiated management of conifer plantations on Mount Lu.

To address these limitations, we used an integrated, paired, cross-plantation experimental design. Permanent plots in Japanese cedar (>*Cryptomeria japonica*) and Hinoki cypress (*Chamaecyparis obtusa*) plantations were sampled before and approximately 12 months after control, light-, moderate-, and heavy-thinning treatments. We simultaneously quantified Hedley P fractions, bacterial and fungal alpha-diversity indices, and potential phosphatase activities in the 0–10 and 10–20 cm soil layers. The distinctive contribution of this study therefore lies in combining paired pre- and post-thinning measurements with a common thinning-intensity gradient across two plantation types and two soil depths, rather than in demonstrating for the first time that thinning can affect soil P. This design allowed us to determine whether short-term thinning-related changes were concentrated in labile and moderately labile P pools, whether these responses depended on plantation type and soil depth, and whether changes in active P fractions were statistically associated with microbial and enzymatic indicators. This study addressed three questions: (1) whether different thinning intensities alter labile and moderately labile P fractions in the short term; (2) whether soil P fractions and microbial responses differ between Japanese cedar and Hinoki cypress plantations; and (3) whether thinning-related changes in P fractions are statistically associated with changes in microbial alpha diversity and phosphatase activity. We hypothesized that: (1) LP and MLP would exhibit significant sampling time × thinning treatment interactions, indicating that their changes from before to after thinning differed among thinning treatments, whereas HRP and RP would show weaker or nonsignificant interactions; (2) thinning effects would differ between soil depths, as indicated by significant thinning treatment × soil depth interactions, with larger changes expected in the 0–10 cm layer than in the 10–20 cm layer; and (3) thinning effects would differ between plantation types, as indicated by significant thinning treatment × plantation type interactions. We further expected changes in LP and MLP to be positively associated with changes in microbial alpha-diversity indices and potential phosphatase activities.

## 2 Materials and methods

### 2.1 Study area

The study was conducted adjacent to the Lushan Botanical Garden in the Lushan National Nature Reserve, Jiujiang City, Jiangxi Province, China (29°32′58″ N, 115°58′54″ E; approximately 1,100 m a.s.l.), where plantations of Japanese cedar (*Cryptomeria japonica*) and Hinoki cypress (*Chamaecyparis obtusa*) are widely distributed. The area has typical montane forest ecological conditions and a subtropical humid monsoon climate with distinct mountain-climate characteristics. The climate is mild and humid, with abundant precipitation, a mean annual temperature of approximately 11.6 °C, annual precipitation of approximately 2060 mm, frequent fog, and high air humidity, all of which favor forest vegetation growth and soil organic matter accumulation.

### 2.2 Thinning experiment design

Japanese cedar (*Cryptomeria japonica*) and Hinoki cypress (*Chamaecyparis obtusa*) plantations were used as the experimental stands. Within each plantation type, candidate plots with comparable initial stand density, stand age, elevation, slope, aspect, and management history were selected. The four treatments—control (CK), light thinning (LT), moderate thinning (MT), and heavy thinning (HT)—were randomly assigned to the selected plots. Control plots were not thinned. The treatments were classified as light thinning (LT; 10% ≤ intensity < 25%), moderate thinning (MT; 25% ≤ intensity < 35%), and heavy thinning (HT; 35% ≤ intensity ≤ 45%). For each plantation type, three plots were assigned to CK and four plots were assigned to each thinning treatment, resulting in 30 permanent plots. Each 30 m × 30 m plot was treated as an independent experimental unit. A 10-m-wide buffer zone was maintained around each plot to minimize edge effects and interference among treatments. The plots were located at an elevation of approximately 1,100 m a.s.l., on slopes of approximately 30°, with predominantly south-facing aspects. Treatments were randomly assigned to plots within each plantation type. Although the plots occurred under broadly comparable site and management conditions, some differences in pre-thinning stand density, mean tree height, and diameter at breast height were present among treatments (**Tables 1**, **2**). These characteristics are therefore reported descriptively and were considered when interpreting the thinning-related responses.

**Table 1:** Stand characteristics and achieved thinning intensities in Hinoki cypress plantations.

Treatment	Before thinning stand density (trees/ha)	After thinning stand density (trees/ha)	Thinning intensity %	Mean tree height m	Mean DBH cm	Understory cover %
HT	1274.07 ± 239.68	760.18 ± 45.16	42.9 ± 5.00	14.82 ± 1.98	20.27 ± 2.5	39.86 ± 8.20
MT	877.78 ± 285.66	626.85 ± 269.66	28.7 ± 1.20	14.82 ± 1.98	19.39 ± 1.03	36.93 ± 3.19
LT	994.45 ± 221.59	759.26 ± 212.08	22.0 ± 1.70	13.87 ± 1.06	18.77 ± 0.85	36.64 ± 4.01
CK	1048.15 ± 227.13	1048.15 ± 227.13	0	16.47 ± 0.94	22.19 ± 1.16	36.83 ± 5.28

CK, control, LT, light thinning, MT,moderate thinning, HT,heavy thinning, DBH,diameter at breast height.

**Table 2:** Stand characteristics and achieved thinning intensities in Japanese cedar plantations.

Treatment	Before thinning stand density (trees/ha)	After thinning stand density (trees/ha)	Thinning intensity %	Mean tree height m	Mean DBH cm	Understory cover %
HT	1011.11 ± 133.28	561.11 ± 70.60	42.0 ± 1.70	13.76 ± 1.16	18.73 ± 1.44	37.63 ± 5.76
MT	801.39 ± 263.17	516.67 ± 181.85	29.6 ± 2.20	14.07 ± 1.46	18.94 ± 1.72	37.15 ± 6.70
LT	731.94 ± 165.87	556.25 ± 70.86	16.0 ± 5.10	13.71 ± 1.03	18.57 ± 1.02	37.79 ± 4.86
CK	933.33 ± 69.43	933.33 ± 69.43	0	16.38 ± 0.90	21.92 ± 1.47	36.93 ± 3.55

### 2.3 Soil sampling and measurements

Baseline soil sampling was conducted in July 2024 immediately before the thinning treatments were implemented. Follow-up soil sampling was conducted in August 2025 in the same permanent plots, approximately 12 months after thinning. In each plot, soil samples were collected from the 0–10 and 10–20 cm layers using a five-point composite sampling method. The five subsamples from the same plot and soil layer were thoroughly mixed to form one independent sample. Each soil sample was divided into separate subsamples immediately after collection. Samples intended for microbial-community analysis were transported to the laboratory in insulated containers with ice packs within 6 h and stored at −80 °C until DNA extraction. Samples used for phosphatase-activity measurements were stored at -20 °C and analyzed within 72 h of collection. No sample underwent repeated freeze-thaw cycles before analysis. The remaining soil was air-dried and used for physicochemical-property and P-fraction analyses.

Soil pH was determined using a potentiometric method. Soil organic carbon (SOC) was measured using the potassium dichromate external-heating method. Total nitrogen (TN) was measured using a continuous-flow analyzer after digestion with concentrated H_2_SO_4_. Ammonium nitrogen (NH_4_^+^-N) and nitrate nitrogen (NO_3_^--^N) were measured using an automated continuous-flow analyzer. Total phosphorus (TP) and available phosphorus (AP) were determined following standard soil agrochemical analysis procedures. Soil P fractions were determined using the Hedley sequential extraction method (Hedley et al., 1982), with reference to modified methods (Tiessen and Moir, 1993; Cross and Schlesinger, 1995). Based on their operationally defined extractability and lability, H_2_O-Pi, NaHCO_3_-Pi, and NaHCO_3_-Po were grouped as labile P (LP); NaOH-Pi and NaOH-Po as moderately labile P (MLP); HCl-Pi and HCl-Po as highly resistant P (HRP); and residual P as residual P (RP). Inorganic P in each extract was measured by the molybdenum blue colorimetric method. Organic P was calculated as the difference between total P and inorganic P in each fraction. Residual P was measured after H_2_SO_4_-H_2_O_2_ digestion. Soil phosphatase activity was determined using the p-nitrophenyl phosphate method. Genomic DNA was extracted from the soil samples before PCR amplification. The V3–V4 region of the bacterial 16S rRNA gene was amplified using primers 341F (5′-CCTACGGGNGGCWGCAG-3′) and 806R (5′-GGACTACHVGGGTATCTAAT-3′), producing an expected amplicon of approximately 466 bp. The fungal ITS2 region was amplified using primers ITS3_KYO2 (5′-GATGAAGAACGYAGYRAA-3′) and ITS4 (5′-TCCTCCGCTTATTGATATGC-3′), producing an expected amplicon of approximately 381 bp.PCR products were recovered by gel purification and quantified using a QuantiFluor fluorometer. The purified amplicons were pooled in equimolar amounts, ligated to sequencing adapters, and used for sequencing-library construction. Paired-end 250-bp sequencing was performed on an Illumina platform by Guangzhou Genedenovo Biotechnology Co., Ltd. (Guangzhou, China). Initial quality processing of the Illumina reads was performed using fastp version 0.18.0. Subsequent sequence processing was conducted using DADA2 and included read filtering, paired-end merging, dereplication, denoising, and chimera removal. Amplicon sequence variants (ASVs) were generated from the denoised, nonchimeric sequences. Taxonomic assignment was performed using the naïve Bayesian algorithm implemented in the RDP Classifier, with a confidence threshold of 0.80. Alpha-diversity indices, including Chao1 and Shannon indices, were calculated from the resulting ASV tables, and rarefaction curves were used to evaluate sequencing-depth sufficiency. The archived sequencing reports did not specify the DNA-extraction kit, numerical DNA-quality acceptance criteria, detailed PCR reaction and thermal-cycling conditions, experimental control configuration, library-preparation kit, exact Illumina instrument model, DADA2 version and filtering parameters, or the name and version of the taxonomic reference database. These unrecorded details were therefore not reconstructed retrospectively (Walters et al., 2016).

### 2.4 Data analysis

Statistical analyses were performed using R version 4.2.1 and SPSS. For each plot and soil layer, the change in each soil variable was calculated as:ΔX=X_post_−X_pre_ where ΔX represents the within-plot change, X_pre_ is the value measured before thinning, and X_post_ is the value measured approximately 12 months after thinning in the same plot and soil layer. The same change-score calculation was applied consistently to all variables and treatment groups. Statistical tests of baseline differences were not used to determine the subsequent analytical method, because a nonsignificant baseline comparison does not demonstrate baseline equivalence.

Treatment effects were evaluated using a factorial repeated-measures analysis of variance to account for the factorial and repeated structure of the experiment. Plantation type and thinning treatment were treated as between-plot factors, whereas sampling time and soil depth were treated as within-plot factors. Each permanent plot was specified as the repeated-measures subject. The model included the main effects of plantation type, thinning treatment, sampling time, and soil depth, together with their interaction terms. The prespecified interactions of primary interest were sampling time × thinning treatment, thinning treatment × soil depth, and plantation type × thinning treatment. Higher-order interactions were also tested to determine whether thinning responses depended simultaneously on plantation type, sampling time, and soil depth. When a significant interaction was detected, simple-effects analyses were conducted within the corresponding factor combinations, followed by Tukey-adjusted pairwise comparisons. Normality and homogeneity of variance were evaluated using model residuals, and statistical significance was accepted at P<0.05. For descriptive presentation and RDA, within-plot changes were calculated as ΔX=X_post_−X_pre_. These change scores were used to describe the direction and magnitude of temporal responses but were not analyzed using separate one-way ANOVAs as independent tests of thinning effects. For conciseness, **Table 3** presents only statistically significant interaction terms; nonsignificant prespecified interactions are described in the Results section.

**Table 3:** Significance tests of interaction effects among stand type, thinning treatment, sampling time, and soil depth on soil phosphorus fractions.

Soil P fraction	Significant interaction	df (numerator, denominator)	F value	P value
LP	S×Y	1, 66	6.780	0.011*
	T×Y	3, 66	6.042	0.001**
	S×D	1, 66	10.066	0.002**
	T×D	3, 66	4.851	0.004**
	S×T×D	3, 66	12.983	<0.001***
MLP	S×D	1, 66	83.104	<0.001***
	T×D	3, 66	10.131	<0.001***
	S×T×D	3, 66	4.705	0.005**
HRP	S×D	1, 66	6.474	0.013*
	T×D	3, 66	8.560	<0.001***
RP	S×D	1, 66	9.725	0.003**
	T×D	3, 66	4.065	0.010*
	S×T×D	3, 66	4.311	0.008**

Factor abbreviations: S, stand type; T, thinning intensity; Y, sampling time (pre- vs. post-thinning); D, soil depth (0–10 cm and 10–20 cm). LP, labile phosphorus; MLP, moderately labile phosphorus; HRP, highly resistant phosphorus; RP, residual phosphorus. *, **, and *** indicate P < 0.05, P < 0.01, and P < 0.001, respectively.

Redundancy analysis (RDA) was conducted separately for each plantation type and soil depth using the same plot-level changes calculated as X_post_−X_pre_ (n=15 plots per ordination). The response matrix comprised bacterial and fungal alpha-diversity indices and acid and alkaline phosphatase activities, whereas the explanatory matrix comprised soil physicochemical properties and P fractions. Variables with strongly skewed distributions were transformed as appropriate, and all variables were standardized before analysis. Candidate explanatory variables were screened for multicollinearity, and variables with variance inflation factors greater than 10 were excluded. The significance of the overall RDA model and the retained explanatory variables was evaluated using 9,999 permutation tests.

## 3 Results

### 3.1 Effects of thinning on soil P fractions in Hinoki cypress and Japanese cedar plantations

Soil P fractions were analyzed using the factorial repeated-measures ANOVA. Significant interactions were followed by simple-effects analyses and Tukey-adjusted pairwise comparisons. Plot-level changes calculated as post-thinning minus pre-thinning values are presented below to describe the direction and magnitude of the responses. Overall, the response was concentrated in the labile P pool and the moderately labile P pool, whereas the highly resistant P pool and residual P showed smaller and less consistent treatment-related changes. Significant stand type × thinning treatment × soil depth interactions for LP and MLP indicated that the magnitude of the response depended jointly on plantation type and soil depth.

In Hinoki cypress plantations, the summed ΔLP values in the 0–10 cm layer were 1.36, 7.75, 15.43, and 15.03 mg kg^-1^ under CK, LT, MT, and HT, respectively, while the corresponding ΔMLP values were 8.47, 21.52, 38.11, and 45.38 mg kg^-1^. Relative to CK, MT and HT increased ΔLP by 14.07 and 13.67 mg kg^-1^ and ΔMLP by 29.64 and 36.91 mg kg^-1^, respectively. Among the component fractions, ΔNaOH-Pi showed the clearest treatment gradient, increasing from 3.75 mg kg^-1^ under CK to 13.49, 26.79, and 35.49 mg kg^-1^ under LT, MT, and HT, with all four treatments assigned to different significance groups. ΔH2O-P was also significantly higher under all thinning treatments than under CK; for ΔNaHCO3-Pi, HT and MT formed the highest significance group, LT was intermediate, and CK was lowest. In the 10–20 cm layer, ΔLP was 2.44, 3.94, 8.91, and 8.44 mg kg^-1^ and ΔMLP was 3.95, 14.59, 21.57, and 29.82 mg kg^-1^ under CK, LT, MT, and HT, respectively. The subsurface response was again driven mainly by ΔNaOH-Pi, which increased stepwise from 2.10 mg kg^-1^ under CK to 6.19, 15.67, and 23.01 mg kg^-1^ under LT, MT, and HT, respectively.

In Japanese cedar plantations, thinning produced a stronger and more consistent increase in active P fractions. In the 0–10 cm layer, ΔLP increased from 5.70 mg kg^-1^ under CK to 13.67, 20.49, and 32.11 mg kg^-1^ under LT, MT, and HT, respectively; ΔMLP increased from 12.49 to 28.20, 35.49, and 53.67 mg kg^-1^. Thus, relative to CK, HT increased ΔLP and ΔMLP by 26.41 and 41.18 mg kg^-1^, respectively. All three LP component fractions increased significantly and progressively with thinning intensity. For the MLP components, HT had the highest ΔNaOH-Pi (32.53 mg kg^-1^) and ΔNaOH-Po (21.14 mg kg^-1^), MT and LT were intermediate, and CK had the lowest values. In the 10–20 cm layer, ΔLP was 7.43, 10.42, 15.43, and 17.41 mg kg^-1^ and ΔMLP was 6.44, 13.04, 18.84, and 25.74 mg kg^-1^ under CK, LT, MT, and HT, respectively. Compared with CK, HT increased ΔLP by 9.98 mg kg^-1^ and ΔMLP by 19.30 mg kg^-1^. Significant treatment differences at this depth were evident for ΔNaHCO3-Pi, ΔNaHCO3-Po, ΔNaOH-Pi, and ΔNaOH-Po, whereas ΔH2O-P did not differ significantly among treatments.

**Figure 1:**
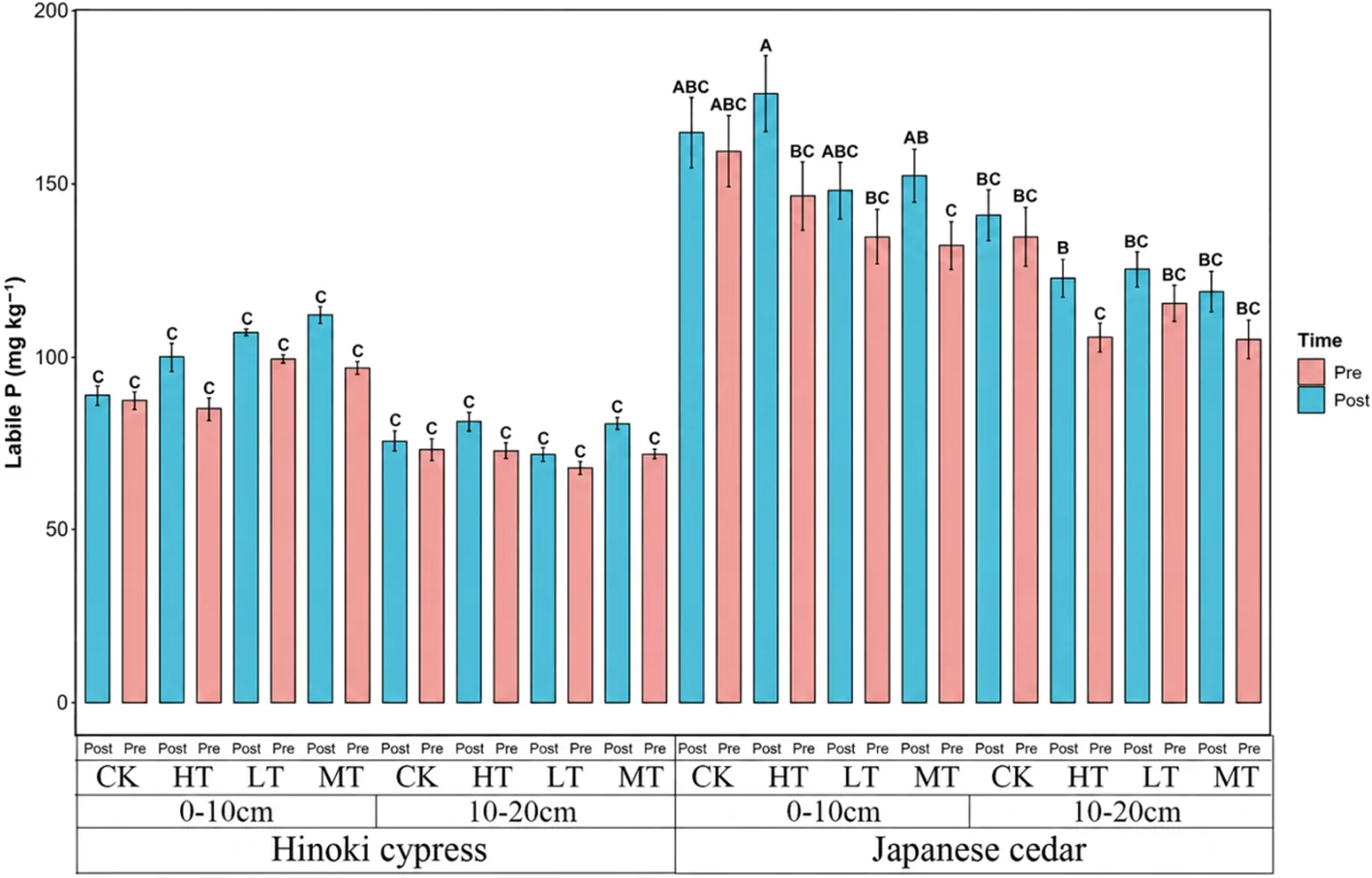
Responses of soil labile phosphorus (P) concentrations to thinning treatments across tree species, sampling times, and soil depths. Bars represent mean ± SE. Different letters indicate significant differences among treatments according to Tukey’s HSD test (p < 0.05). LP, labile P; MLP, moderately labile P; HRP, highly resistant P; RP, residual P. CK, control; LT, light thinning; MT, moderate thinning; HT, heavy thinning.

Across both plantation types, treatment-related increases were generally greater in the 0–10 cm layer than in the 10–20 cm layer, and Japanese cedar showed the largest overall increases, particularly under HT. Within each soil layer, ΔHCl-Pi, ΔHCl-Po, and ΔRP did not show consistent significant differences among thinning treatments despite some numerical variation. These results indicate that short-term thinning primarily redistributed P toward labile and moderately labile pools rather than consistently altering highly resistant or residual P pools (**Tables 4**, **5**; **Figure 1**).

**Table 4:** Incremental changes in soil phosphorus fractions in Hinoki cypress plantations under different thinning treatments.

Soil depth(cm)	Treatment	ΔH2O-P (mg kg^-1^)	ΔNaHCO3-Pi (mg kg^-1^)	ΔNaHCO3-Po (mg kg^-1^)	ΔNaOH-Pi (mg kg^-1^)	ΔNaOH-Po (mg kg^-1^)	ΔHCl-Pi (mg kg^-1^)	ΔHCl-Po (mg kg^-1^)	ΔResidual-P (mg kg^-1^)
0-10	HT	3.70 ± 0.57a	8.07 ± 0.92a	3.26 ± 0.98ab	35.49 ± 1.62a	9.89 ± 3.15a	7.68 ± 10.62a	5.23 ± 1.21a	5.30 ± 1.87a
	MT	3.02 ± 0.27a	6.90 ± 1.41a	5.51 ± 0.97a	26.79 ± 4.91b	11.32 ± 5.29a	7.66 ± 9.37a	5.80 ± 2.36a	4.31 ± 2.74a
	LT	1.85 ± 0.43a	2.64 ± 1.10b	3.26 ± 1.94ab	13.49 ± 5.69c	8.03 ± 5.63a	9.91 ± 3.85a	1.01 ± 2.57a	8.34 ± 3.41a
	CK	0.38 ± 0.57b	0.68 ± 0.60c	0.30 ± 0.98c	3.75 ± 1.77d	4.72 ± 3.54a	3.37 ± 1.31a	3.46 ± 5.15a	6.25 ± 2.17a
10-20	HT	2.28 ± 0.58A	3.75 ± 1.11A	2.41 ± 0.25AB	23.01 ± 3.98A	6.81 ± 1.39A	5.05 ± 7.28A	0.85 ± 0.51A	3.59 ± 4.53A
	MT	2.58 ± 0.16A	3.81 ± 0.40A	2.52 ± 1.09A	15.67 ± 4.34B	5.90 ± 6.54A	3.91 ± 3.26A	0.84 ± 0.53A	5.33 ± 3.65A
	LT	1.17 ± 0.21B	2.08 ± 0.58A	0.69 ± 1.13B	6.19 ± 1.13C	8.40 ± 2.40A	5.39 ± 1.14A	6.73 ± 9.31A	6.40 ± 1.78A
	CK	0.66 ± 0.98B	1.01 ± 2.02A	0.77 ± 0.39B	2.10 ± 0.95D	1.85 ± 0.83A	4.77 ± 4.45A	13.64 ± 15.50A	5.90 ± 3.18A

Δ indicates the post-thinning value minus the corresponding pre-thinning value. CK, control; LT, light thinning; MT, moderate thinning; HT, heavy thinning; Pi, inorganic phosphorus; Po, organic phosphorus; H_2_O-P, water-extractable phosphorus; NaHCO_3_-Pi, sodium bicarbonate-extractable inorganic phosphorus; NaHCO_3_-Po, sodium bicarbonate-extractable organic phosphorus; NaOH-Pi, sodium hydroxide-extractable inorganic phosphorus; NaOH-Po, sodium hydroxide-extractable organic phosphorus; HCl-Pi, hydrochloric acid-extractable inorganic phosphorus; HCl-Po, hydrochloric acid-extractable organic phosphorus; Residual-P, residual phosphorus.Values are presented as mean ± standard error. Within each phosphorus fraction, different lowercase letters indicate significant differences among thinning treatments in the 0–10 cm soil layer, whereas different uppercase letters indicate significant differences among thinning treatments in the 10–20 cm soil layer (P < 0.05). Treatments sharing the same letter are not significantly different. The letters are not intended for comparisons between soil depths.

**Table 5:** Incremental changes in soil phosphorus fractions in Japanese cedar plantations under different thinning treatments.

Soil depth (cm)	Treatment	ΔH2O-P (mg kg^-1^)	ΔNaHCO3-Pi (mg kg^-1^)	ΔNaHCO3-Po (mg kg^-1^)	ΔNaOH-Pi (mg kg^-1^)	ΔNaOH-Po (mg kg^-1^)	ΔHCl-Pi (mg kg^-1^)	ΔHCl-Po (mg kg^-1^)	ΔResidual-P (mg kg^-1^)
0-10	HT	7.65 ± 1.02a	11.49 ± 1.35a	12.97 ± 2.71a	32.53 ± 4.88a	21.14 ± 3.87a	12.22 ± 3.42a	11.36 ± 2.58a	16.61 ± 9.98a
	MT	5.24 ± 0.75b	7.40 ± 0.85b	7.85 ± 1.22b	21.14 ± 2.56b	14.35 ± 2.19b	7.24 ± 1.51a	7.05 ± 0.49a	14.80 ± 5.15a
	LT	3.28 ± 0.55c	5.02 ± 0.84c	5.37 ± 0.77c	15.07 ± 2.60b	13.13 ± 1.72b	10.10 ± 3.26a	7.81 ± 5.02a	16.08 ± 3.05a
	CK	1.83 ± 0.34d	1.89 ± 0.25d	1.98 ± 0.51d	6.36 ± 7.85c	6.13 ± 1.75c	10.36 ± 3.50a	6.96 ± 3.99a	13.71 ± 5.68a
10-20	HT	4.35 ± 0.71A	6.20 ± 0.84A	6.86 ± 1.28A	19.71 ± 3.30A	6.03 ± 1.26A	9.33 ± 2.44A	6.64 ± 2.00A	13.67 ± 5.39A
	MT	4.27 ± 1.11A	4.62 ± 0.91B	6.54 ± 1.59B	13.92 ± 1.90B	4.92 ± 1.35B	8.23 ± 3.05A	4.29 ± 0.12A	12.71 ± 5.25A
	LT	3.69 ± 1.24A	3.49 ± 0.64B	3.24 ± 0.86C	9.19 ± 1.88C	3.85 ± 0.60B	6.54 ± 3.51A	4.30 ± 2.05A	13.03 ± 6.32A
	CK	3.21 ± 3.46A	1.73 ± 0.22C	2.49 ± 1.28D	3.81 ± 0.60D	2.63 ± 0.53B	5.10 ± 5.41A	5.68 ± 4.97A	12.25 ± 2.91A

### 3.2 Effects of thinning on soil microbial alpha diversity and phosphatase activity

The effects of thinning on microbial alpha diversity and phosphatase activity varied among plantation types and thinning intensities. Microbial alpha-diversity indices and phosphatase activities were evaluated within the factorial repeated-measures framework. Where significant interactions were detected, treatment-specific differences were examined using simple-effects analyses and Tukey-adjusted pairwise comparisons within the corresponding plantation type, sampling time, and soil depth.

**Figure 2:**
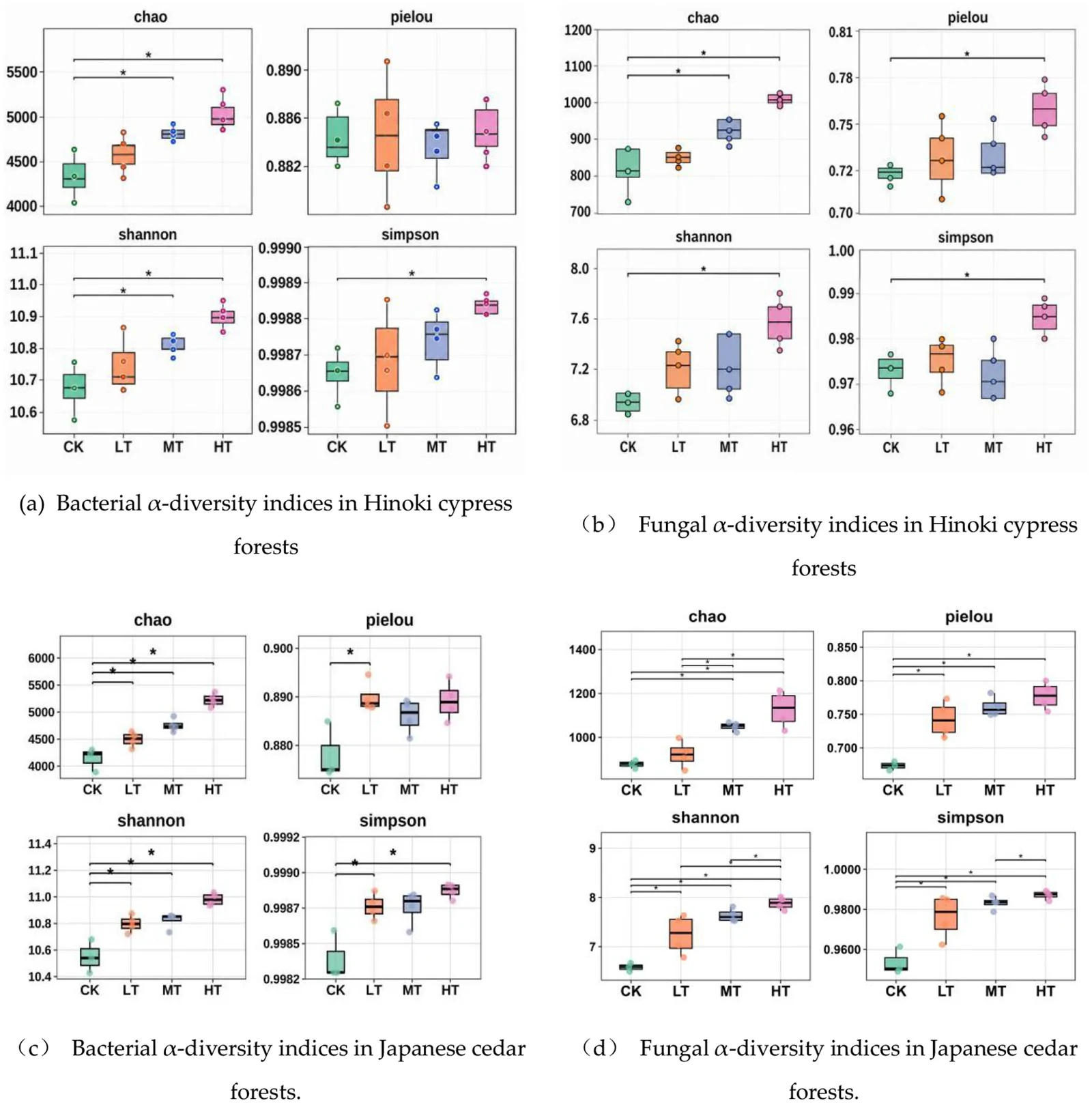
Bacterial and fungal alpha-diversity indices under different thinning treatments in Hinoki cypress and Japanese cedar plantations. Panels show **(a)** bacterial alpha diversity in Hinoki cypress plantations, **(b)** fungal alpha diversity in Hinoki cypress plantations, **(c)** bacterial alpha diversity in Japanese cedar plantations, and **(d)** fungal alpha diversity in Japanese cedar plantations. The horizontal line within each box represents the median, the lower and upper boundaries represent the first and third quartiles, and the whiskers extend to observations within 1.5 times the interquartile range. Points represent individual plot-level observations. Within each plantation type, CK contained three independent plots, whereas LT, MT, and HT each contained four independent plots. For each alpha-diversity index, treatment effects were evaluated using a two-way analysis of variance, with thinning treatment, plantation type, and their interaction as fixed factors; bacterial and fungal datasets were analyzed separately. When a significant interaction or main effect was detected, simple-effects and pairwise comparisons of estimated marginal means were conducted with Tukey adjustment. Horizontal brackets indicate the treatment pairs being compared, and asterisks indicate significant pairwise differences (adjusted P < 0.05).

In Hinoki cypress plantations, bacterial and fungal alpha diversity after thinning was generally higher than that under CK (**Figure 2**; **Table 6**). The bacterial Chao and Shannon indices reached relatively high levels under HT, and the Shannon index under MT and HT was significantly higher than under CK. For the fungal community, the Chao and Shannon indices under HT were significantly higher than those under CK. fungal Chao and Shannon indices under HT were higher than those under CK, indicating that relatively high-intensity thinning increased soil fungal richness and diversity in Hinoki cypress plantations. Acid phosphatase responded more strongly to thinning than alkaline phosphatase. In the 0–10 cm layer, acid phosphatase activity under MT and HT was significantly higher than under CK and LT, whereas the response in the 10–20 cm layer was weaker.

**Table 6:** Effects of thinning intensity on soil bacterial and fungal α-diversity in Hinoki cypress and Japanese cedar forests.

Community	Treatment	n	Chao	Simpson	Pielou evenness	Shannon
Fungal Community of Hinoki cypress	CK	3	828.33 ± 49.99b	0.972484 ± 0.004075b	0.7119 ± 0.0049b	6.8980 ± 0.0658b
	LT	4	851.33 ± 20.23ab	0.973396 ± 0.007283ab	0.7250 ± 0.0293ab	7.1589 ± 0.2632ab
	MT	4	931.00 ± 47.32a	0.969809 ± 0.009311ab	0.7291 ± 0.0211ab	7.2596 ± 0.4625ab
	HT	4	1015.33 ± 10.41a	0.984754 ± 0.004676a	0.7576 ± 0.0221a	7.5672 ± 0.2317a
BActerial Community of JHinoki cypress	CK	3	4336.17 ± 266.91b	0.998651 ± 0.000070b	0.8847 ± 0.0024a	10.6869 ± 0.0937b
	LT	4	5036.00 ± 171.61ab	0.998830 ± 0.000033ab	0.8854 ± 0.0030a	10.8881 ± 0.0321ab
	MT	4	4548.33 ± 234.45a	0.998686 ± 0.000176ab	0.8846 ± 0.0060a	10.7480 ± 0.1019a
	HT	4	4825.67 ± 17.95a	0.998734 ± 0.000107a	0.8835 ± 0.0028a	10.8114 ± 0.0374a
BActerial Community of Japanese Cedar	CK	3	4138.00 ± 216.83b	0.998395 ± 0.000172b	0.8782 ± 0.0059b	10.5497 ± 0.1259b
	LT	4	4493.00 ± 141.20a	0.998762 ± 0.000091a	0.8899 ± 0.0032a	10.7975 ± 0.0646a
	MT	4	4757.25 ± 122.55a	0.998756 ± 0.000121ab	0.8860 ± 0.0036ab	10.8235 ± 0.0600a
	HT	4	5223.25 ± 127.03a	0.998872 ± 0.000052a	0.8892 ± 0.0041ab	10.9814 ± 0.0466a
Fungal Community of Japanese Cedar	CK	3	877.33 ± 18.56b	0.953602 ± 0.006760c	0.6733 ± 0.0068b	6.5826 ± 0.0866c
	LT	4	921.75 ± 60.62b	0.976364 ± 0.011066ab	0.7427 ± 0.0268a	7.2431 ± 0.4070b
	MT	4	1048.50 ± 19.91a	0.983201 ± 0.003291b	0.7610 ± 0.0148a	7.6359 ± 0.1338b
	HT	4	1128.25 ± 83.07a	0.987113 ± 0.002174a	0.7775 ± 0.0205a	7.8802 ± 0.1257a

CK, control; LT, light thinning; MT, moderate thinning; HT, heavy thinning; n, number of independent plots.

**Figure 3:**
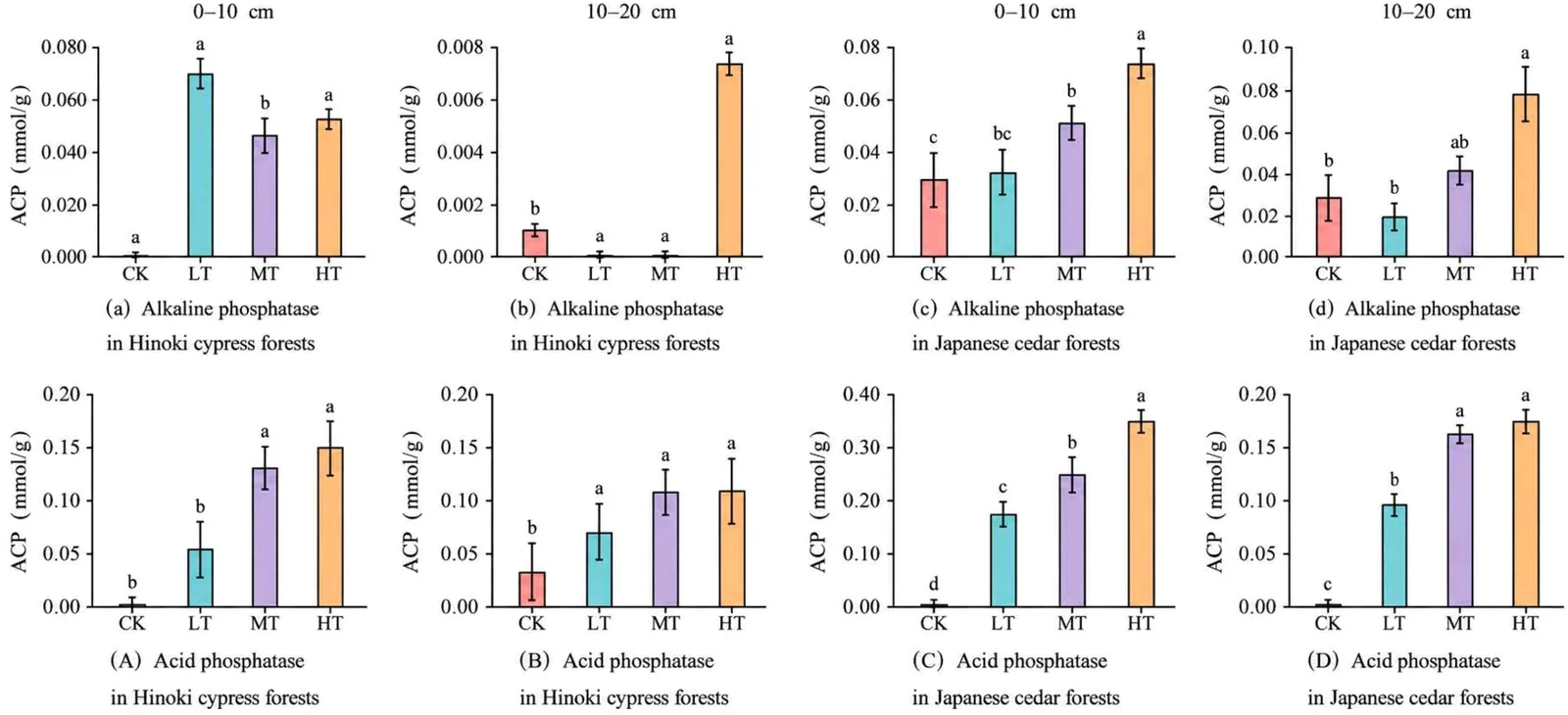
Changes in potential soil alkaline phosphatase (ALP) and acid phosphatase (ACP) activities under different thinning treatments in Hinoki cypress and Japanese cedar plantations. Changes were calculated for each plot as the post-thinning value minus the corresponding pre-thinning value. Panels show **(a)** ALP activity in the 0–10 cm soil layer of Hinoki cypress plantations, **(b)** ALP activity in the 10–20 cm soil layer of Hinoki cypress plantations, **(c)** ALP activity in the 0–10 cm soil layer of Japanese cedar plantations, **(d)** ALP activity in the 10–20 cm soil layer of Japanese cedar plantations, **(A)** ACP activity in the 0–10 cm soil layer of Hinoki cypress plantations, **(B)** ACP activity in the 10–20 cm soil layer of Hinoki cypress plantations, **(C)** ACP activity in the 0–10 cm soil layer of Japanese cedar plantations, and **(D)** ACP activity in the 10–20 cm soil layer of Japanese cedar plantations. Bars represent means, and error bars represent standard deviations. Within each plantation type, CK contained three independent plots, whereas LT, MT, and HT each contained four independent plots. For each enzyme and soil depth, changes were analyzed using a two-way ANOVA with thinning treatment, plantation type, and their interaction as fixed factors. Following a significant interaction or main effect, simple-effects comparisons among thinning treatments within each plantation type were conducted using Tukey-adjusted pairwise tests. Different lowercase letters indicate significant differences among thinning treatments within the same plantation type, soil depth, and enzyme (adjusted P < 0.05); treatments sharing the same letter do not differ significantly. Enzyme activity is expressed as μmol g^-1^ d^-1^.

In Japanese cedar plantations, several bacterial and fungal alpha-diversity indices were numerically higher under thinning than under CK, with the highest mean values generally observed under HT; however, the magnitude and statistical significance of the responses varied among indices. The bacterial Chao index increased from approximately 4150 under CK to approximately 5200 under HT, and the Shannon index increased from approximately 10.55 to 11.00. The fungal Chao index increased from approximately 880 under CK to approximately 1140 under HT, and the Shannon index increased from approximately 6.60 to 7.90, indicating that fungal communities in Japanese cedar plantations were more sensitive to thinning. Potential phosphatase activities also showed treatment-specific changes, with acid phosphatase generally showing larger numerical responses than alkaline phosphatase. acid phosphatase activity in the 0–10 cm layer increased from approximately 0.01 under CK to 0.35 mg phenol g^-^¹ h^-^¹1 under HT and from approximately 0.01 to 0.20 mg phenol g^-^¹ h^-^¹ in the 10–20 cm layer. Alkaline phosphatase activity showed smaller and less consistent treatment-related changes than acid phosphatase activity (**Figure 3**).

Overall, microbial alpha-diversity indices and phosphatase activities showed treatment-specific responses to thinning. Several microbial indices and acid phosphatase activities were higher under moderate or heavy thinning than under CK, but the responses were not consistently monotonic or statistically significant across all indicators, plantation types, and soil depths. Changes in acid phosphatase activity co-occurred with changes in LP and MLP, indicating an association between potential phosphatase activity and active soil P pools. However, these results do not directly demonstrate microbially mediated organic P mineralization.

### 3.3 Relationships among soil microbial diversity, phosphatase activity, and soil P fractions

**Figure 4:**
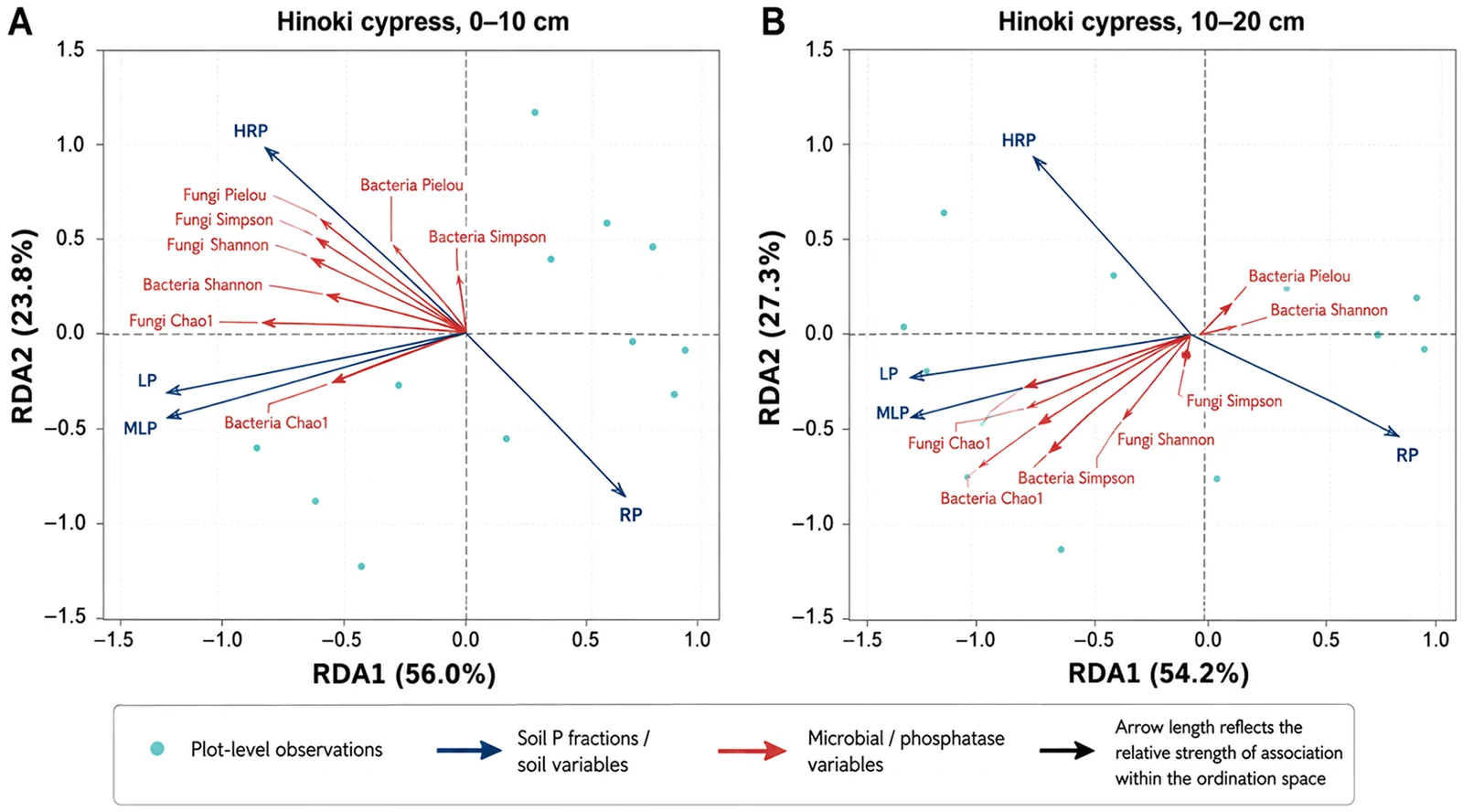
Redundancy analysis of changes in soil P fractions, soil physicochemical variables, microbial alpha-diversity indices, and potential phosphatase activities in Hinoki cypress plantations at 0–10 and 10–20 cm soil depths. Light-teal points represent plot-level observations. Dark-blue arrows represent soil P fractions and soil physicochemical variables, whereas red arrows represent microbial alpha-diversity indices and potential phosphatase activities. Arrow direction indicates the direction of association within the displayed ordination space, and arrow length represents the relative strength of association within each panel. RDA1 and RDA2 percentages indicate the proportions of constrained variation explained by the corresponding axes. Arrow lengths should not be quantitatively compared across panels. **(A)** represents the 0–10 cm soil depth, and **(B)** represents the 10–20 cm soil depth.

**Figure 5:**
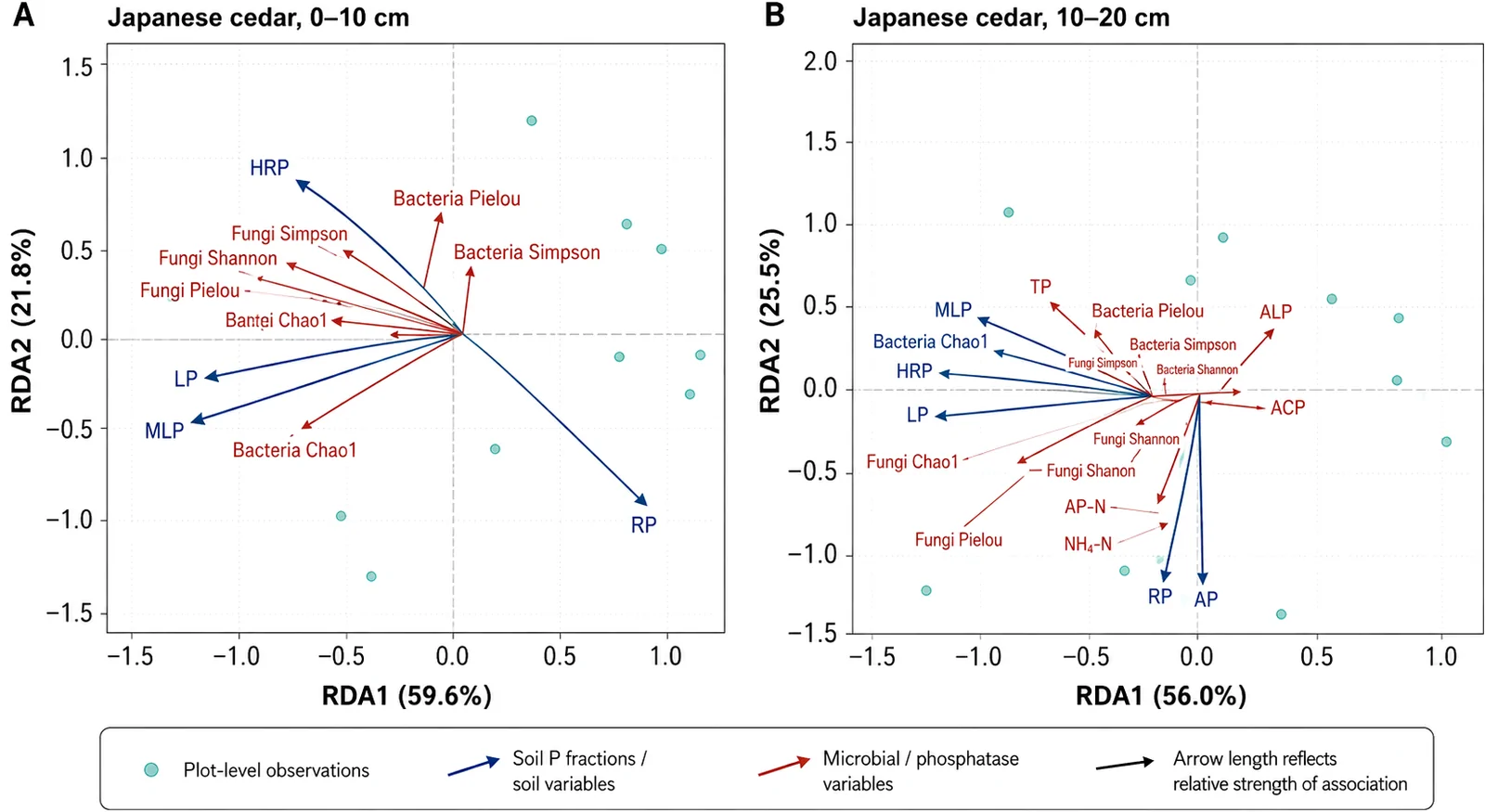
Redundancy analysis of changes in soil P fractions, soil physicochemical variables, microbial alpha-diversity indices, and potential phosphatase activities in Japanese cedar plantations at 0–10 and 10–20 cm soil depths. Light-teal points represent plot-level observations. Dark-blue arrows represent soil P fractions and soil physicochemical variables, whereas red arrows represent microbial alpha-diversity indices and potential phosphatase activities. Arrow direction indicates the direction of association within the displayed ordination space, and arrow length represents the relative strength of association within each panel. RDA1 and RDA2 percentages indicate the proportions of constrained variation explained by the corresponding axes. Arrow lengths should not be quantitatively compared across panels. **(A)** represents the 0–10 cm soil depth, and **(B)** represents the 10–20 cm soil depth.

RDA indicated strong associations among soil P fractions, microbial alpha diversity, and phosphatase activity, and these associations were more pronounced in Japanese cedar plantations and in the 0–10 cm soil layer (**Figures 4**, **5**). The first two ordination axes cumulatively explained more than 75% of the variation, indicating that the ordination captured the relationships between soil P fractions and microbial indicators well. In the ordination space, LP, MLP, and AP were oriented in the same direction as most bacterial and fungal alpha-diversity indices and phosphatase activities, indicating close relationships between labile and moderately labile P fractions and microbial responses. In contrast, In the RDA ordination, the RP vector was oriented in the opposite direction to most microbial alpha-diversity and phosphatase variables, indicating a negative association within the displayed ordination space. In Japanese cedar plantations, LP, MLP, and AP clustered more closely with microbial alpha diversity and phosphatase activity, further indicating stronger coupling between changes in soil P availability and microbial processes.

In summary, changes in soil P fractions after thinning were characterized mainly by increases in LP and MLP and were consistent with enhanced microbial alpha diversity and phosphatase activity. These results indicate that short-term increases in surface-soil P availability were closely associated with changes in microbial alpha diversity and phosphatase activity, particularly in Japanese cedar plantations.

## 4 Discussion

### 4.1 Thinning improved soil P availability mainly by increasing labile and moderately labile P fractions

This study showed that soil P fractions in Japanese cedar and Hinoki cypress plantations on Mount Lu changed markedly after thinning, but the changes were concentrated mainly in LP and MLP, whereas HRP and RP showed relatively weak responses. This indicates that short-term thinning did not primarily increase stable P pools but may have improved soil P availability by promoting the activation of transformable P pools. LP generally represents P pools that can be directly used or readily transformed by plants and microorganisms, whereas MLP is often associated with Fe/Al oxide-bound P and some organic P and serves as an important potential source of available P in acidic forest soils. Our findings broadly agree with previous studies in Larix principis-rupprechtii and Pinus massoniana plantations showing that thinning can alter labile and moderately labile P fractions. However, the direction and magnitude of these responses have varied among studies depending on thinning intensity, soil depth, plantation type, and recovery period (Tian et al., 2019; Liu et al., 2018; Jian et al., 2025; Ye et al., 2018). Compared with these variable responses, the present study showed relatively consistent increases in LP and MLP, particularly in the 0–10 cm soil layer and in Japanese cedar plantations under heavy thinning. The stronger surface-soil response may reflect rapid changes in litter inputs, fine-root turnover, rhizosphere activity, and microbial processes following canopy opening. Differences among studies may also be attributable to tree-species traits, initial stand density, soil properties, thinning intensity, sampling depth, and the time elapsed since treatment (Hou et al., 2020; Jian et al., 2025; Rocha et al., 2019; Xu et al., 2022).

This response was concentrated mainly in the 0–10 cm surface layer, indicating that surface soil is a key interface through which thinning affects P cycling. Thinning reduced stand density and increased canopy openness, thereby improving understory light, hydrothermal conditions, and the environment for understory vegetation development. These changes may have promoted litter decomposition, fine-root turnover, and rhizosphere activity. Because surface soil directly receives litter and root residues, it is particularly sensitive to stand-structure regulation. In contrast, stable P fractions such as HCl-P and residual P have slower turnover rates and are less likely to respond significantly to thinning in the short term, which explains the weak responses of HRP and RP observed in this study (Jian et al., 2025; Rocha et al., 2019).

### 4.2 Microbial diversity and phosphatase activity may be important biological pathways through which thinning affects P transformation

Taken together, the treatment-related changes in microbial alpha diversity and acid phosphatase activity, together with their associations with active P fractions in the RDA, are consistent with a potential microbe–enzyme–P availability linkage following thinning (Fierer, 2017; Philippot et al., 2024). By improving the forest microenvironment and increasing organic substrate inputs, thinning provides additional niches and energy sources for soil microorganisms, thereby favoring increases in microbial richness and diversity. Bacteria usually respond rapidly to changes in readily available carbon sources, nitrogen, and available P, whereas fungi play important roles in decomposing complex organic matter, such as lignin and cellulose, and in organic P mineralization. The coordinated changes in microbial alpha diversity, acid phosphatase activity, and active P fractions are consistent with a potential microbial–enzymatic contribution to soil P transformation. Although plant roots can also release acid phosphatase, microbial communities are important potential contributors to acid phosphatase activity in bulk non-rhizosphere soil. Therefore, the observed associations among microbial alpha diversity, acid phosphatase activity, and active P fractions support a plausible microbe-enzyme-P availability linkage following thinning. However, microbial alpha diversity reflects community richness and evenness rather than the abundance or expression of P-cycling functional genes. Therefore, increased alpha diversity alone cannot demonstrate enhanced organic matter decomposition, phosphatase production, or P-mineralization capacity (Frąc et al., 2018; Bahram et al., 2018; Crowther et al., 2019; Delgado-Baquerizo et al., 2016).

This interpretation is further supported by the stronger response of acid phosphatase than alkaline phosphatase under the acidic soil conditions of the conifer plantations on Mount Lu. Potential acid phosphatase activity was associated with active P fractions, but the bulk-soil assay did not distinguish microbial from root-derived enzymes or quantify *in situ* P mineralization. Thus, the increase in potential acid phosphatase activity was consistent with an enhanced potential capacity for organic P mineralization. However, because *in situ* mineralization was not measured, its contribution to the observed changes in active P fractions cannot be quantified or confirmed. Several limitations should be acknowledged. This study identified concurrent changes in soil P fractions, microbial alpha diversity, and potential phosphatase activity, but it did not directly test the mechanisms underlying these relationships. Microbial alpha diversity is not a direct measure of P-cycling function, and the bulk-soil phosphatase assay neither distinguished microbial enzymes from root-derived enzymes nor quantified *in situ* organic P mineralization. Therefore, the proposed microbe–enzyme–P availability linkage should be regarded as a mechanistic hypothesis supported by statistical associations rather than as a directly demonstrated causal pathway. In addition, Fe/Al oxide forms, P sorption-desorption characteristics, and rhizosphere organic acid concentrations were not measured. Therefore, the observed changes in NaOH-extractable P should be interpreted only as changes in an operationally defined P fraction, rather than as direct evidence for Fe/Al-bound P mobilization. The proposed Fe/Al-associated pathway should consequently be regarded as an untested hypothesis requiring direct experimental verification.

Taken together, the concurrent changes in microbial alpha-diversity indices, potential phosphatase activity, and active P fractions indicate statistical associations among these variables. These associations are ecologically consistent with a possible microbe–enzyme–P availability linkage, but they do not establish the direction of influence or demonstrate a causal mediation pathway.

### 4.3 Potential microbial and phosphatase-related pathways associated with thinning-induced changes in soil P availability

The observed changes in soil P availability after thinning were closely associated with microbial alpha-diversity indices and phosphatase activity, suggesting a potential contribution of microbial and enzymatic processes to soil P transformation (Tedersoo et al., 2020; Trivedi et al., 2020). By reducing stand density, thinning improved understory light, hydrothermal conditions, and the growth environment of understory vegetation, which may have promoted litter decomposition, fine-root turnover, and organic matter inputs. These changes can provide additional carbon sources and nutrient substrates for soil microorganisms. Increased bacterial and fungal alpha diversity may enhance organic matter decomposition and organic P mineralization. Meanwhile, the marked increase in acid phosphatase activity suggests that this enzyme may represent an important pathway for converting organic P into available P after thinning. Acid phosphatase may contribute to the mineralization of organic P contained within operationally defined fractions such as NaHCO_3_-Po and NaOH-Po, potentially releasing inorganic P and contributing to increases in labile and moderately labile P pools (Jian et al., 2025).

One possible, but untested, explanation is that thinning-related changes in root and microbial activity altered the production of organic acids and other metabolites, which could potentially affect P sorption-desorption processes in acidic soils. The observed increase in NaOH-extractable P is consistent with an accumulation or redistribution of P within an operationally defined moderately labile pool. However, NaOH-extractable P is not a direct measure of Fe/Al-bound P mobilization, and its increase cannot demonstrate that Fe/Al-bound P was released or transformed. Because Fe/Al oxide forms, P sorption-desorption characteristics, and rhizosphere organic acid concentrations were not measured, the proposed Fe/Al-associated pathway remains an untested hypothesis requiring direct experimental verification.

### 4.4 Species differences indicate that thinning effects are regulated by stand characteristics

The significant plantation type × thinning treatment × soil depth interactions for LP and MLP indicated that thinning-response patterns differed between plantation types and soil depths. In Japanese cedar plantations, the largest increases in active P fractions occurred under heavy thinning, whereas in Hinoki cypress plantations, increases were evident under both moderate and heavy thinning. These contrasting treatment-specific patterns may be related to differences in canopy structure, litter quality, root distribution, understory vegetation recovery, and microbial substrate supply between the two plantation types. The treatment-specific increases observed in Japanese cedar plantations under heavy thinning may indicate that stand-structure adjustment more readily created a soil microenvironment favorable for litter decomposition and microbial activity (Liu et al., 2023; Xu et al., 2024; Zhang et al., 2023).

In contrast, LP and MLP in Hinoki cypress plantations also increased with thinning intensity, but the overall response magnitude was lower, and both moderate and heavy thinning showed positive effects. This suggests that plantation species differ in sensitivity to thinning intensity and that a single thinning standard should not be applied uniformly. For Japanese cedar plantations, higher-intensity thinning was associated with larger short-term changes in some active P fractions in the studied stands. However, these results do not establish an optimal thinning intensity because tree growth, regeneration, plant P uptake, P losses, and long-term soil responses were not evaluated. Longer-term monitoring is required before management recommendations can be made. However, because this study reflects only short-term effects one year after thinning, long-term nutrient loss, P-pool depletion, and microbial community re-stabilization require further monitoring.

In Japanese cedar plantations, microbial indicators clustered more closely with LP, MLP, AP, and nutrient factors, suggesting stronger coupling among soil nutrient availability, P transformation, and microbial responses. This stronger coupling may partly explain the greater sensitivity of Japanese cedar plantations to thinning.

## 5 Conclusion

One year after thinning, the observed changes in soil P were concentrated mainly in labile and moderately labile fractions, whereas highly resistant and residual P fractions showed comparatively limited responses. Changes in active P fractions co-occurred with variation in microbial alpha diversity and potential phosphatase activity, indicating statistical associations rather than demonstrated microbial or enzymatic mechanisms. These findings suggest that thinning may promote short-term redistribution of soil P among operationally defined fractions. However, longer-term monitoring of soil P stocks, plant P uptake, microbial functional traits, and P sorption-desorption processes is required before an optimal thinning intensity can be recommended.

## Data Availability

The raw 16S rRNA gene and ITS amplicon sequencing data generated in this study have been deposited in the Genome Sequence Archive (GSA) at the National Genomics Data Center, China National Center for Bioinformation, under accession number CRA048172 (BioProject: PRJCA071594).
